# Mathematical method to build an empirical model for inhaled anesthetic agent wash-in

**DOI:** 10.1186/1471-2253-11-13

**Published:** 2011-06-24

**Authors:** Jan FA Hendrickx, Harry Lemmens, Sofie De Cooman, André AJ Van Zundert, René EJ Grouls, Eric Mortier, Andre M De Wolf

**Affiliations:** 1 Department of Anesthesiology, Intensive Care and Pain Therapy, Onze Lieve Vrouwziekenhuis, Aalst, Belgium; 2 Department of Anesthesia, Stanford School of Medicine, Stanford, California, USA; 3 Department of Anesthesiology, Sint-Jan Hospital, Brussels, Belgium; 4 Department of Anesthesiology, Intensive Care and Pain Therapy, Catharina Hospital, Eindhoven, The Netherlands; 5 Department of Clinical Pharmacy, Catharina Hospital, Eindhoven, The Netherlands; 6 Department of Anesthesiology, University of Ghent, Ghent, Belgium; 7 Department of Anesthesiology, Northwestern University Medical School, Chicago, Illinois, USA

## Abstract

**Background:**

The wide range of fresh gas flow - vaporizer setting (FGF - F_D_) combinations used by different anesthesiologists during the wash-in period of inhaled anesthetics indicates that the selection of FGF and F_D _is based on habit and personal experience. An empirical model could rationalize FGF - F_D _selection during wash-in.

**Methods:**

During model derivation, 50 ASA PS I-II patients received desflurane in O_2 _with an ADU^® ^anesthesia machine with a random combination of a fixed FGF - F_D _setting. The resulting course of the end-expired desflurane concentration (F_A_) was modeled with Excel Solver, with patient age, height, and weight as covariates; NONMEM was used to check for parsimony. The resulting equation was solved for F_D_, and prospectively tested by having the formula calculate F_D _to be used by the anesthesiologist after randomly selecting a FGF, a target F_A _(F_At_), and a specified time interval (1 - 5 min) after turning on the vaporizer after which F_At _had to be reached. The following targets were tested: desflurane F_At _3.5% after 3.5 min (n = 40), 5% after 5 min (n = 37), and 6% after 4.5 min (n = 37).

**Results:**

Solving the equation derived during model development for F_D _yields F_D_=-(e^(-FGF*-0.23+FGF*0.24)^*(e^(FGF*-0.23)^*F_At_*Ht*0.1-e^(FGF*-0.23)^*FGF*2.55+40.46-e^(FGF*-0.23)^*40.46+e^(FGF*-0.23+Time/-4.08)^*40.46-e^(Time/-4.08)^*40.46))/((-1+e^(FGF*0.24)^)*(-1+e^(Time/-4.08)^)*39.29). Only height (Ht) could be withheld as a significant covariate. Median performance error and median absolute performance error were -2.9 and 7.0% in the 3.5% after 3.5 min group, -3.4 and 11.4% in the 5% after 5 min group, and -16.2 and 16.2% in the 6% after 4.5 min groups, respectively.

**Conclusions:**

An empirical model can be used to predict the FGF - F_D _combinations that attain a target end-expired anesthetic agent concentration with clinically acceptable accuracy within the first 5 min of the start of administration. The sequences are easily calculated in an Excel file and simple to use (one fixed FGF - F_D _setting), and will minimize agent consumption and reduce pollution by allowing to determine the lowest possible FGF that can be used. Different anesthesia machines will likely have different equations for different agents.

## Background

What fresh gas flow - vaporizer setting (FGF - F_D_) combination should be used for a particular patient when starting the administration of potent inhaled anesthetics to reach a target end-expired concentration (F_A_) after a predetermined time interval without excessively wasting potent inhaled anesthetic? The wide range of FGF - F_D _combinations used by different anesthesiologists during the wash-in period of potent inhaled anesthetics indicates that the selection of FGF and F_D _is based on habit and personal experience. Some anesthesiologists use a high FGF (to shorten the wash-in time constant of the anesthesia circle breathing system, and to avoid rebreathing that results in dilution of F_D_), while others prefer to use a lower FGF in combination with a higher F_D _(to compensate for the longer wash-in time constant, and to reduce agent consumption). While all anesthesiologists swiftly attain the target F_A _(F_At_) because FGF and F_D _can be adjusted according to the measured F_A_, it is unlikely that the particular FGF - F_D _combination used was that with the least number of F_D _and FGF adjustments and minimum waste. The use of high FGF, even for a seemingly brief period (5 min), may increase agent consumption above that of an ensuing one hour maintenance phase with a 1 L. min^-1 ^FGF [1], and may forfeit the savings of an automated closed-circuit anesthesia machine [2].

Instead of relying on personal preference, we hypothesize that very specific FGF - F_D _combinations can be used in the individual patient to attain a F_At _within a specified time interval by using an empirical model of the kinetics of inhaled anesthetics during wash-in. Simple, easy to remember FGF - F_D _combinations construed from these models could reduce agent consumption while not distracting the anesthesiologist from other tasks during the induction period of anesthesia [3].

While kinetics of inhaled anesthetics in the anesthesia circle system have already been modeled using mass balances [4-6], few of these models have been tested prospectively [3,7]. We developed an empirical model for desflurane administered in O_2 _with an ADU - AS/5^® ^anesthesia machine (Anesthesia Delivery Unit, General Electric, Helsinki, Finland) during the first 5 min of the anesthetic, and prospectively tested whether it allows the anesthesiologist to select a FGF - F_D _combination to attain a F_At _of the inhaled anesthetic within a specified time interval (1 - 5 min) after turning on the vaporizer.

## Methods

### Part I. Model development

After obtaining IRB approval (OLV Hospital, Aalst, Belgium) and written informed consent, 50 ASA physical status I or II patients presenting for plastic, urologic, or gynecologic surgery were enrolled. All patients received oral alprazolam (0.5 or 1.0 mg) 1 h before the scheduled start of surgery. After preoxygenation (8 L. min^-1 ^O_2 _FGF for 3 min), propofol (3 mg. kg^-1^), rocuronium (0.7 mg. kg^-1^), and sufentanil (0.1 μg. kg^-1^) were administered intravenously. After tracheal intubation, ventilation was mechanically controlled by an ADU anesthesia machine. Tidal volume and respiratory rate were set fixed at 500 mL and 10 breaths. min^-1^, respectively.

In a particular patient, one (fixed) O_2 _FGF and desflurane F_D _combination was used, with the FGF ranging from 0.5 tot 5 L. min^-1 ^and F_D _from 6 to 18%; this FGF - F_D _combination was chosen randomly (random function in excel). Preliminary trials indicated what combinations were likely to lead to a desflurane F_A _less than 1% after 2 min or less than 2% after 5 min, and these were not considered.

Inspired and expired gases were analyzed by a multigas analyzer (Datex-Ohmeda Compact Airway Module M-CAiOV^®^, Datex-Ohmeda, Helsinki, Finland) and downloaded into a spreadsheet every 10 seconds. Gases were sampled at the distal end of the endotracheal tube using a piece of sampling tubing placed through an Arndt Multi-Port Airway Adapter^® ^(Cook Medical Inc., Bloomington, IN). Gases sampled by the gas analyzer were redirected to the anesthesia circuit via the expiratory limb. The study was terminated after 5 minutes, or earlier when the end-expired desflurane concentration had reached 8%. All values above 8% were eliminated from further analysis. The 5 min period was somewhat arbitrarily defined as the wash-in period because it encompasses (1) anesthesia circuit wash-in; (2) FRC wash-in; (3) early uptake by the VRG; (4) and the waning effects of propofol after about 5 min.

**Figure 1:**
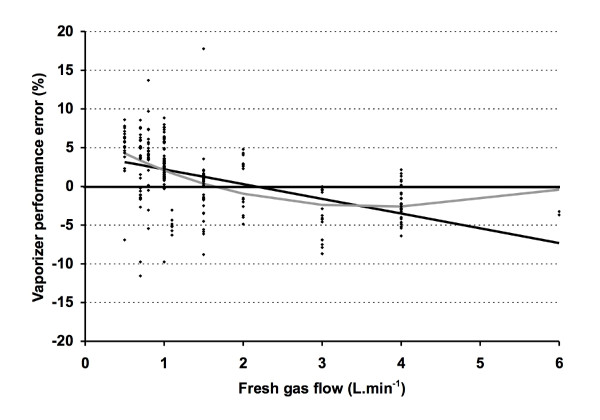
**Vaporizer performance error**. Vaporizer performance error (PE) with different fresh gas flows (FGF). PE (%) = 100*(measured output - vaporizer dial)/vaporizer dialed. Thick line = linear regression, with PE = 4.101-1.902*FGF (r^2 ^= 0.23); grey line = non-linear regression (third order polynomial), with PE = 6.990 - 5.959*FGF + 1.099*FGF^2 ^- 0.05192*FGF^3^.

All measurements were done with the same anesthesia machine and gas analyzer. The ADU^® ^circle system volume is 3.4 L. The fresh gas flow inlet is located distal to the inspiratory valve. Fresh gas flow compensation is used to compensate for the inspiratory fresh gas flow during inspiration. The vaporizer output was measured at the common gas outlet by the same gas analyzer and compared with the dial setting using linear and non-linear regression because (1) the vaporizer output may not match the dial setting; (2) we wanted to be able to generalize the results to other ADU^® ^units; and (3) we wanted to exclude that certain performance error patterns could be related to systematic vaporizer error. Based on 229 measurements in 48 patients in this study, the actual desflurane vaporizer output (%) could be described as -0.72 + 1.075*dial setting (r^2 ^= 0.98); the vaporizer's output tended to increase with lower FGF (Figure 1).

A model was build to relate F_A _to FGF, F_D_, time, and the following patient covariates: age, height, and weight. A constant ventilation allowed us to at least standardize circuit and FRC wash-in; after 5 min, ventilation can easily be adjusted to the desired end-expired CO_2 _concentration. All values before 1 min were deleted because zero values are hard to work with mathematically, and because the model was only supposed to model the FGF - F_D _- time relationship between 1 and 5 min. Initial model building and parameter exploration were done in Excel using Solver^® ^(Microsoft, Seattle, WA). The initial choice of mathematical functions was guided by three assumptions. First, because F_A _rises exponentially, a one exponential function was used to describe the rise of F_A_. Second, the effect of a higher F_D _was modeled as curvilinear (assuming, for example, that doubling F_D _would lead to a doubling of F_A_). Third, the effect of FGF was modeled with a single exponential (increasing as FGF is lowered). By trial and error, these and additional functions were added, deleted, modified, etc. to minimize the sum of least squares (difference between measured and predicted F_A_) using Solver (Excel). Residuals were plotted against height, weight, and age to search for covariate effects by visual inspection and by linear regression. Finally, the model was tested for parsimony using NONMEM's Minimum Objective Function (ICON Development Solutions, Dublin, Ireland).

### Part II. Prospective testing

The model equation derived in part I was solved for F_D _using Mathematica (Mathematica for Windows, Version 4.0, Wolfram Research Inc, Champaign, IL). The resulting equation predicts the FGF - F_D _combinations the anesthesiologist can use to attain a F_At _within the same time interval used during model development (i.e., between 1 and 5 min) using a single F_D _and FGF setting, and takes into account the covariate effects derived during model building (see results section for actual equation).

Management of the patients during prospective testing only differed in the manner in which FGF and F_D _were selected. Patients received desflurane in O_2 _with the goal to reach a F_At _of 3.5% after 3.5 min (n = 40), 5% after 5 min (n = 37), or 6% after 4.5 min (n = 37). The number of patients was chosen based on prior experience. After entering the time and F_At _as well as significant patient covariates in the equation, the equation describes all possible FGF - F_D _combinations that reach the F_At _at the desired time for a patient with the particular characteristics entered into the equation. The fixed FGF that was going to be used in the individual patient was randomly selected (using Excel's random function) and entered in the equation, yielding the F_D _to be used. Because the resolution of the desflurane vaporizer is 0.5%, the nearest value was chosen. FGF values requiring an F_D _above the vaporizer limit (18%) obviously could not be tested.

To allow us to compare model performance between the three subgroups (3.5% after 3.5 min, 5% after 5 min, or 6% after 4.5 min), the performance error (PE) for each patient was calculated as 100*((F_A _measured - F_A _predicted)/F_A _predicted), and the absolute performance error (APE) as the absolute value of PE. Next, for each subgroup, the following were examined: (1) bias and accuracy, using the median performance error (MDPE, median of all PE) and median absolute performance error (MDAPE, median of all APE) [8]; (2) the relationship between FGF and PE (and APE) using linear regression (linear correlation) and a third order polynomial (non-linear effects) to help assess whether the model systematically over- or underestimated the end-expired desflurane concentration with increasing FGF.

## Results

### Part I. Model derivation

Patient demographics are presented in Table 1. The following equation described measured F_A _best:

**Table 1:** Patient demographics, presented as mean (standard deviation)

Group	Number	Age (years)	Weight (kg)	Height (cm)
Model derivation group	50	52 (15)	70 (14)	166 (9)
3.5% after 3.5 min group	40	46 (16)	72 (12)	170 (9)
5% after 5 min group	37	53 (18)	77 (16)	170 (8)
6% after 4.5 min group	37	52 (16)	73 (14)	169 (9)

with Ht = height (cm), Time = time after turning on the vaporizer (min), and with F_A_, F_D _and FGF expressed in %, %, and L. min^-1^, respectively. The measured versus model predicted F_A _are presented in Figure 2. Only height decreased the NONMEM minimum objective function significantly, and therefore is the only patient covariate to be incorporated into the model. Plots of the residuals versus patient covariates are presented in Figure 3. Note that the rebreathing function (1-e^(-FGF*-0.23)^+2.49*(1-e^(-FGF*0.24)^)*0.39*F_D_), will cause the F_A _to decrease again at high flows, an effect caused by a decreased vaporizer output with high FGF due to cooling.

**Figure 2:**
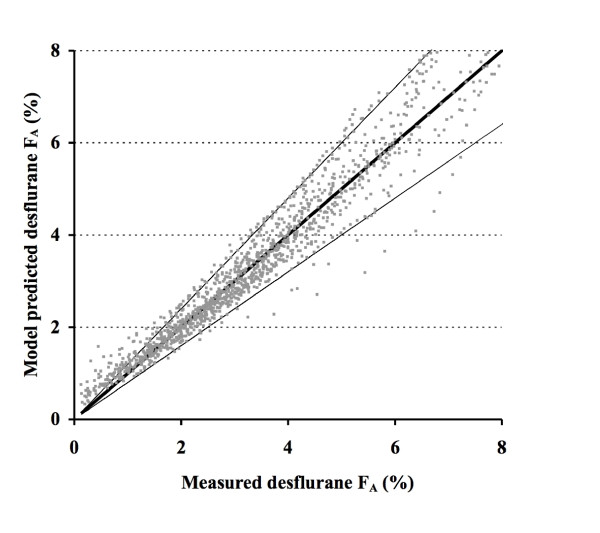
**Model derivation: measured versus model predicted end-expired desflurane concentrations (F**_**A**_**, %)**. Thick line = identity line; lines above and below are + or - 20% deviation from identity line.

**Figure 3:**
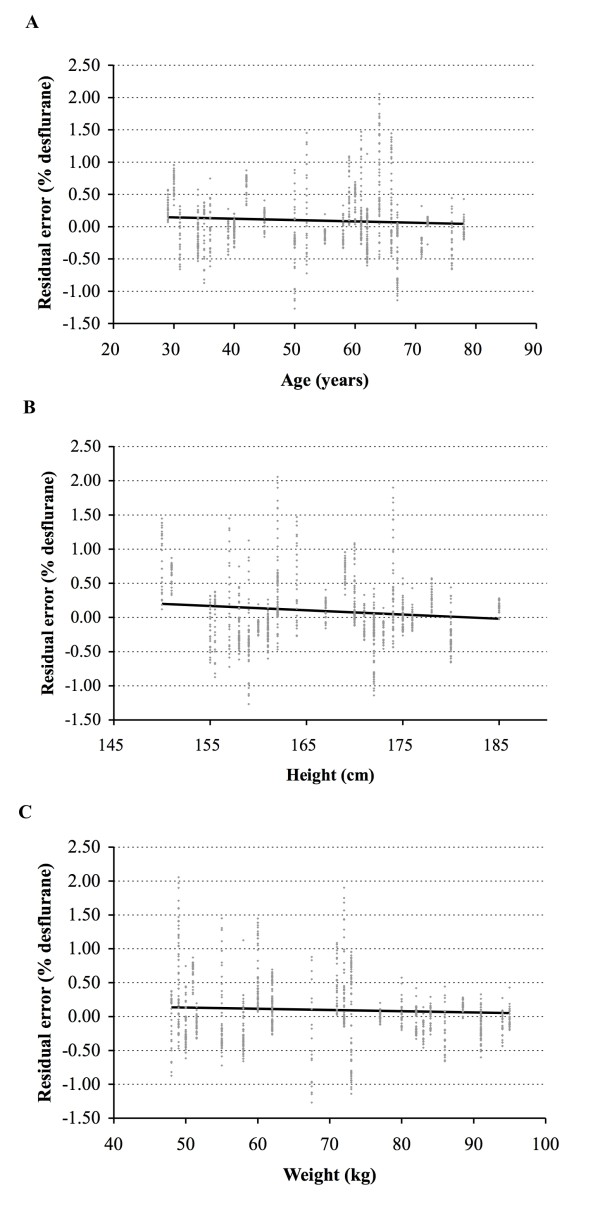
**Model derivation: final plot of residual error (modeled minus measured F**_**A**_**) versus patient covariates**. A = age, B = height, and C = weight. There was no (linear) correlation with age (r^2 ^= 0.00), height (r^2 ^= 0.01 after implementing it as a covariate in the model) and weight (r^2 ^= 0.00).

### Part II. Prospective testing

**Figure 4:**
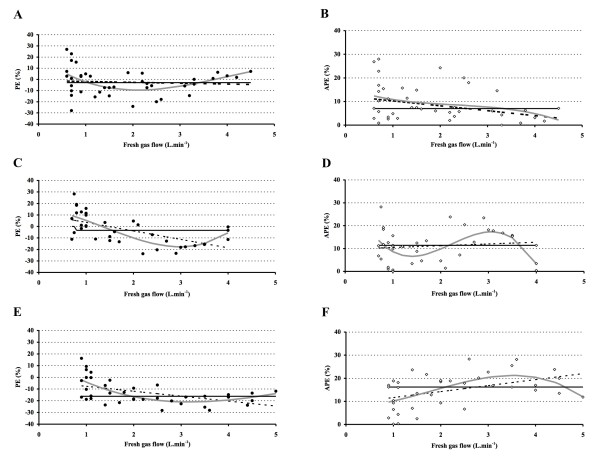
**Model performance**. Performance Error (PE, %, closed circles, group 3.5% after 3.5 min = A; group 5% after 5 min = C, and group 6% after 4 min = E) and Absolute Performance Error (APE, %, open circles, group 3.5% after 3.5 min = B, group 5% after 5 min = D, and group 6% after 4 min = F), with their respective linear regression line (hatched), polynomial fit (grey line), and median performance error (MDPE, continuous black line, A, C, and E) and median absolute performance error (MDAPE, continuous black line, B, D, and F).

**Figure 5:**
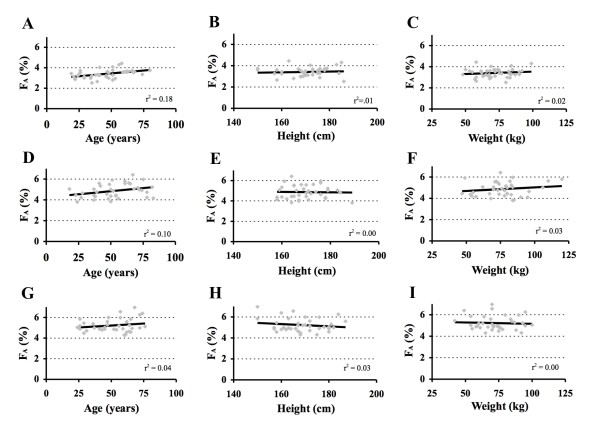
**Prospective testing**. Linear regression between covariates (age, height, and weight) and residual error (predicted minus measured end expired desflurane concentration) in the 3.5% after 3.5 min group (A, B, and C), the 5% after 5 min group (D, E, and F), and the 6% after 4.5 min group (G, H, and I).

Solving the above derived equation for F_D _yields the following equation:

where Time = desired time lag after turning on the vaporizer to reach F_At _(any value between 1 and 5 min); and Ht = patient height in cm. After entering F_At_, Time, Ht, and chosen FGF, the corresponding F_D _to reach F_At _within the desired time lag can be calculated. Because any FGF can be chosen, this will result in a virtually infinite number of FGF - F_D _combinations. Patient demographics for each subgroup are presented in Table 1. Performance parameters PE, MDPE, APE and MDAPE for the three subgroups are presented in Figure 4, that also includes the linear regression line and a third order polynomial fit to examine whether PE and APE systematically increased or decreased with increasing FGF. MDPE and MDAPE were -2.9 and 7.0% in the 3.5% after 3.5 min group, -3.4 and 11.4% in the 5% after 5 min group, and -16.2 and 16.2% in the 6% after 4.5 min groups, respectively. There was a very weak (linear) correlation between F_A _and age, but not with height (effect incorporate in model) and weight (Figure 5).

## Discussion

An empirical equation can be derived that predicts FGF - F_D _combinations that attain a F_At _of an inhaled anesthetic after a predefined time lag. With modern computing power such an equation can easily be entered into an Excel file to calculate the required F_D _for any chosen FGF. While our current model will need to be refined and tested for different patient populations, agents, carrier gases, and anesthesia machines, our results prove the concept.

The concept of using an equation to predict the required F_D _for any chosen FGF to help the anesthesiologist attain and maintain a F_At _is not new. Three decades ago, Lowe described a "general anesthetic equation" that describes the FGF - F_D _relationship in a circle breathing system [4]. That equation was derived by considering mass balances in the circle system and by making certain assumptions regarding the uptake pattern of the agent (the square root of time model) [4]. The model was mainly developed to facilitate the use of closed circuit anesthesia, and has never been tested prospectively across the entire FGF spectrum. Our current empirical model only describes the first 5 min of inhaled agent administration, the wash-in phase, and is to be combined (in the future) with a model that predicts the FGF - F_D _relationship during the maintenance phase.

The MDPE (-2.9, -3.4, and -16.2%) as well as the MAPDE (7.0, 11.4, and 16.2%) are within the limits deemed acceptable for target controlled infusion systems for intravenous anesthetic agents (MDPE <10-20% and MDAPE 20 - 40%) [9]. Still, there is some degree of misspecification of the model that could not be accounted for: there are some outliers of 30%, and the error seems larger in the lower FGF range. The latter could be explained by the fact that uptake differs almost 170% among patients [10,11]. This variability in uptake may have a more pronounced effect on F_A _with lower FGF because increased rebreathing causes the effect of the (unpredictable) amount of uptake on the composition of the inspired mixture to become more pronounced. During modeling, attempts are made to improve the degree of misspecification by taking the effect of covariates into account. However, only height significantly decreased the minimum objective function. Weight (within the range encountered in the study population) has previously been shown not to correlate with agent uptake [10-13]. Therefore, it is no surprise that weight did not improve the minimum objective function during model development, and that there was no significant effect of weight as a covariate. Nevertheless, future modeling in a still larger patient group might reveal for example that lean body weight might be a useful covariate. Age did not improve NONMEMs minimum objective function during model development either, but visual inspection of the F_A _versus age plot during prospective testing suggests F_A _to be higher with older age (r^2 ^ranged from 0.04 - 0.18). Age might therefore still turn out to be a useful covariate in a new model based on a larger number of patients. Other factors may be important - it may be that model misspecification increases with altered physiology and pathophysiology, e.g. when older, sicker patients are included (patients in this study were relatively healthy - ASA 1-2), when cardiac output is altered, or when synergistic effects are likely to come into play (e.g. with premedication).

Other limitations exist. The wash-in model may not be applicable when spontaneous ventilation is allowed immediately following intravenous induction of anesthesia, because the irregular and inconsistent breathing at that time does not allow the acquisition of reliable end-expired concentrations. This also is an issue for modern anesthesia machines that use automated closed-loop end-tidal feedback administration of inhaled agents. Also, the model is limited to a maximum end-expired concentration of 8% - higher concentrations might be needed in some patients, but this comes at a risk of irritating the airway.

**Figure 6:**
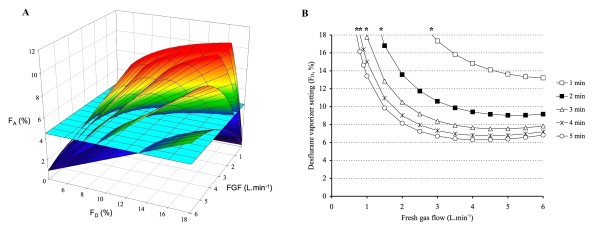
**Visual presentation of model-based desflurane F**_**A **_**course in a fictive 176 cm tall patient**. The multi-colored graphs (A) describe the predicted F_A _resulting from different FGF - F_D _combinations after 1, 2, 3, 4, and 5 min (bottom to top, respectively). The intersection of these surfaces with the F_At _surface, in this example 4.5% desflurane (light blue horizontal surface), yields the corresponding FGF - F_D _combinations that attain that F_At _(B). The asterixes (*) indicate the lowest possible FGF that can be used to attain 4.5% desflurane at 1, 2, 3, 4, and 5 min because of the maximum 18% vaporizer setting. More details are provided in the Appendix.

An empirical equation that describes the FGF - F_D _relationship may have some interesting applications. It can be used to predict and depict the course of F_A _in the individual patient over a wide range of FGF - F_D _settings during the first 5 min of an anesthetic. In figure 6A, the equation has been solved for a 176 cm tall patient to describe the F_A _resulting from different FGF - F_D _combinations after 1, 2, 3, 4, and 5 min (multi-colored graphs, bottom to top, respectively; more detail on how this graph was derived can be found in the Appendix). The equation can further be used to calculate the FGF - F_D _combinations that attain a F_At _after a predefined time interval. If the F_At _would be 4.5% (the light blue surface), the required FGF - F_D _combinations that attain 4.5% after 1, 2, 3, 4, and 5 min can be found by calculating the intersection between the multicolored surfaces and the light blue surface. These intersections are presented in Figure 6B. This figure also illustrates how the equation can be used to calculate the lowest FGF that could be used with the maximum 18% vaporizer setting to attain a certain F_At _(i.e., it illustrates the FGF below which vaporizer output becomes inadequate to attain the F_At _within the specified time interval).

## Conclusion

An empirical model is described that allows the derivation of a formula for each type of anesthesia machine that predicts the FGF - F_D _combinations in a circle breathing system that attain a target end-expired agent concentration in a particular patient within a specified time interval (1 - 5 min) after turning on the vaporizer. The sequences are easily calculated in an Excel file and simple to use (one fixed FGF - F_D _setting), and have the potential to minimize agent consumption and reduce pollution by allowing to determine the lowest possible FGF that can be used.

## Appendix: Derivation of Figure 6

According to the model, F_A _= (1/(Ht*0.1))*(2.55*FGF+1348*(1-e^(-FGF*-0.23)^+2.49*(1-e^(-FGF*0.24)^)*0.39*F_D_)*(0.03*(1-e^(Time/-4.08)^))) with Ht = height (cm), Time = time after turning on the vaporizer (min), and with F_A_, F_D _and FGF expressed in %, %, and L. min^-1^, respectively.

**Figure 7:**
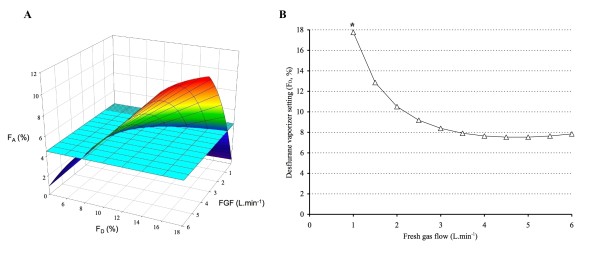
**Multicolored surface describing desflurane F**_**A **_**after 3 min for a 176 cm tall patient over the FGF - F**_**D **_**range studied**. The FGF - F_D _combinations that result in a F_At _of 4.5% (light blue horizontal surface) after 3 min are found by calculating the intersection between this target (the light blue surface) and the multicolored surface; these FGF - F_D _combinations are presented in B. The asterix (*) indicates the lowest possible FGF that can be used to attain 4.5% desflurane at 3 min because of the maximum 18% vaporizer setting.

Let us examine how the desflurane F_A _would evolve for a 176 cm tall patient over the entire FGF-F_D _range studied during model development. The surface describing desflurane F_A _after 3 min is calculated by entering the following parameters into the above formula: Ht = 176; Time = 3; and a sufficient number of FGF - F_D _combinations to allow the reconstruction of a surface in Sigmaplot (Systat Software Inc, San Jose, CA, USA). The multicolored surface in Figure 7 represents these predicted F_A _values after 3 min.

**Figure 8:**
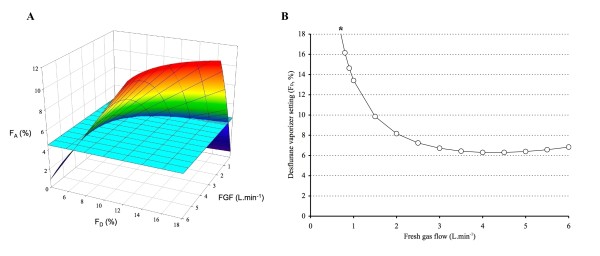
**Multicolored surface describing desflurane F**_**A **_**after 5 min for a 176 cm tall patient over the FGF - F**_**D **_**range studied**. The FGF - F_D _combinations that result in a F_At _of 4.5% (light blue surface) after 5 min are found by calculating the intersection between this target (the light blue surface) and the multicolored surface; these FGF - F_D _combinations are presented in B. The asterix (*) indicates the lowest possible FGF that can be used to attain 4.5% desflurane at 5 min because of the maximum 18% vaporizer setting.

To determine which FGF - F_D _combinations result in a F_At _of e.g. 4.5% after 3 min, the intersection has to be calculated between this target (the horizontal light blue surface) and the multicolored surface that describes the F_A _values with the entire FGF-F_D _range after 3 min (Figure 7). This line can be calculated by entering Ht = 176, Time = 3, and F_At _= 4.5 in the formula F_D_=-(e^(-FGF*-0.23+FGF*0.24)^*(e^(FGF*-0.23)^*F_At_*Ht*0.1-e^(FGF*-0.23)^*FGF*2.55+40.46-e^(FGF*-0.23)^*40.46+e^(FGF*-0.23+Time/-4.08)^*40.46-e^(Time/-4.08)^*40.46))/((-1+e^(FGF*0.24)^)*(-1+e^(Time/-4.08)^)*39.29). This intersection line is shown in Figure 8 and presents the virtually infinite number of FGF - F_D _combinations that result in an F_A _of 4.5% after 3 min in a 176 cm tall patient. It also illustrates that it is not possible to achieve this target with a FGF lower than 0.7 L. min^-1 ^because F_D _would have to be higher than the maximum 18%. Note that the curve increases at high FGF because vaporizer output decreases due to excessive cooling.

Similar graphs can be developed for different time intervals (between 1 and 5 min). The multicolored surface in Figure 7 describes the desflurane F_A _after using a range of FGF - F_D _combinations for 5 min; the intersection between this surface and the target 4.5% surface (horizontal light blue surface) thus describes the virtually infinite number of FGF - F_D _combinations that result in an F_A _of 4.5% after 5 min in a 176 cm tall patient (Figure 8). Figure 6 is a composite of these graphs for the time intervals of 1, 2, 3, 4, and 5 min with a F_At _of 4.5%.

## Competing interests

Jan Hendrickx has received speaker fees and reimbursement of travel expenses related to presentation of his work from GE, Abbott, and Baxter.

## Authors' contributions

JH, HL and ADW conceptualized the idea, designed the study, and performed mathematical analysis. All authors participated in data analysis and interpretation and drafting of the manuscript. All authors read and approved the final manuscript.

## Pre-publication history

The pre-publication history for this paper can be accessed here:

http://www.biomedcentral.com/1471-2253/11/13/prepub
